# Adaptive Iterated Extended Kalman Filter and Its Application to Autonomous Integrated Navigation for Indoor Robot

**DOI:** 10.1155/2014/138548

**Published:** 2014-02-13

**Authors:** Yuan Xu, Xiyuan Chen, Qinghua Li

**Affiliations:** ^1^School of Instrument Science and Engineering, Southeast University, Nanjing, China; ^2^Key Laboratory of Micro-Inertial Instrument and Advanced Navigation Technology, Ministry of Education, Nanjing, China; ^3^School of Electrical Engineering and Automation, Qilu University of Technology, Jinan, China

## Abstract

As the core of the integrated navigation system, the data fusion algorithm should be designed seriously. In order to improve the accuracy of data fusion, this work proposed an adaptive iterated extended Kalman (AIEKF) which used the noise statistics estimator in the iterated extended Kalman (IEKF), and then AIEKF is used to deal with the nonlinear problem in the inertial navigation systems (INS)/wireless sensors networks (WSNs)-integrated navigation system. Practical test has been done to evaluate the performance of the proposed method. The results show that the proposed method is effective to reduce the mean root-mean-square error (RMSE) of position by about 92.53%, 67.93%, 55.97%, and 30.09% compared with the INS only, WSN, EKF, and IEKF.

## 1. Introduction

As the development of automation indoor mobile robots, how to obtain accurate navigation information of indoor mobile robots has received great attention over the past few decades.

To the integrated system, the global positioning systems (GPS)/inertial navigation systems (INS) integrated system is one of the most used methods for the outdoor navigation. Many attempts try to improve the accuracy of the GPS/INS integration. For example, Quinchia et al. compared different error modeling of MEMS applied to GPS/INS integrated systems in [1], Jwo et al. proposed a fuzzy adaptive strong tracking unscented Kalman filter for ultratight GPS/INS integrated systems [2], Chen et al. proposed a GPS/INS system using novel filtering methods for vessel attitude determination [3], and Jwo et al. proposed a nonlinear filtering with IMM algorithm for ultratight GPS/INS integration [4]. Meanwhile, in order to overcome the GPS outage, some attempts try to design bridge methods by using the artificial intelligence algorithms [5] such as Neural Networks (NN) [6–8] and least squares support vector machine (LS-SVM) [9–11]. However, since the accuracy of the integrated system is depending on the GPS, it has poor performance in the indoor environment. In order to get higher positioning accuracy indoor, some literatures try to employ wireless localization to replace the GPS in the integrated system. For instance, S. J. Kim and B. K. Kim proposed an accurate hybrid global self-localization algorithm for indoor mobile robots with two-dimensional isotropic ultrasonic receivers [12], and an accurate pedestrian indoor navigation by tightly coupling foot-mounted IMU and RFID measurements was proposed in [13]. On the basis of the navigation strategy, the data fusion algorithm should also be designed seriously. In this field, Kalman filter (KF) is able to achieve the optimal state estimation [14]. However, it is not suitable for nonlinear systems. Thus, the extended KF (EKF) is proposed to overcome this problem by Taylor series expansion, which may introduce a truncated error [15]. In order to overcome this problem, the iterated EKF (IEKF) is proposed. However, the data fusion algorithms mentioned above are difficult to track the accurate state during the target's fast movement since it employs a fixed priori estimates for the process and measurement noise covariances during the whole estimation process [16].

In order to overcome these problems, we employed the noise statistics estimator in the IEKF, which combines the advantages of the AEKF and the IEKF. The remainder of the paper is organized as follows. Sections 2 and 3 give the introduction for AIEKF and its application to INS/WSN integrated system. The tests and discussion are illustrated in Section 4. Finally, the conclusions are given.

## 2. Adaptive Iterated Extended Kalman Filter

In this section, a brief introduction to the EKF and IEKF will be given, and then AIEKF will be proposed. It is assumed that a discrete-time model of a nonlinear system is given by

(1) xk=f(xk−1)+ωk,yk=h(xk)+υk,

where **x**_*k*_ and **y**_*k*_ are the state vector and the measurement vector for the filter, *f*(·) and *h*(·) are the dynamic model function and the measurement function, respectively, and *ω*_*k*_ and *υ*_*k*_ are the process noise vector and measurement noise vector, respectively. It is assumed that *ω*_*k*_ and *υ*_*k*_ are independent zero-mean white Gaussian sequences with covariance **Q** and **R**, respectively.

### 2.1. Extended Kalman Filter

The traditional EKF algorithm is utilizing a set of equations as follows [17]:

(2) $$\begin{array}{c} {\hat{X}}_{k|k-1}=A_{k|k-1}{\hat{X}}_{k-1|k-1}+{\hat{q}}_{k}, \end{array}$$

(3) $$\begin{array}{c} P_{k|k-1}=A_{k|k-1}P_{k-1}A_{k|k-1}^{T}+{\hat{Q}}_{k}, \end{array}$$

(4) $$\begin{array}{c} K_{k}=P_{k|k-1}{H_{k}}^{T}{\left[H_{k}P_{k|k-1}{H_{k}}^{T}+{\hat{R}}_{k}\right]}^{-1}, \end{array}$$

(5) $$\begin{array}{c} \upsilon_{k}=y_{k}-h\left({\hat{X}}_{k|k-1}^{}\right), \end{array}$$

(6) $$\begin{array}{c} {\hat{X}}_{k|k}^{}={\hat{X}}_{k|k-1}^{}+K_{k}^{}\upsilon_{k}, \end{array}$$

(7) $$\begin{array}{c} P_{k|k}^{}=\left[I-K_{k}^{}H\left({\hat{X}}_{k|k}^{}\right)\right]P_{k|k-1}, \end{array}$$

where$\;{\;A}_{k|k-1}=\partial f\left({\hat{X}}_{k|k}\right)/\partial {\hat{X}}_{k|k}$, $H_{k}=\partial h\left({\hat{X}}_{k|k}^{}\right)/\partial {\hat{X}}_{k|k}^{}$.

### 2.2. Iterated Extended Kalman Filter

Compared with the EKF, the IEKF employs a few simple iterative operations to reduce the bias and the estimation error after getting  **X**_*k*_  in (2) and  **P**_*k*_  in (3). The corresponding recursive relations are

(8) X^k|k(1)=X^k|k−1,Pk|k(1)=Pk|k−1,Kk(n)=Pk|k−1(H(n))T[H(n)Pk|k−1(H(n))T+R]−1,X^k|k(n+1)=X^k|k(n)+Kk(n)[yk−h(n)(X^k|k(n))−H(n)  ×(X^k|k−1−X^k|k(n))],Pk|k(n)=[I−Kk(n)H(n)]Pk|k−1(n),

where$\;\;H^{\left(n\right)}=\partial h\left({\hat{X}}_{k|k}^{n}\right)/\partial {\hat{X}}_{k|k}^{n}$ and the superscript *n*  (*n* = 1,2,…, *m*)  is the number of iteration steps, And then,

(9) $$\begin{array}{c} {\hat{X}}_{k|k}^{}={\hat{X}}_{k|k}^{\left(m\right)},\\ P_{k|k}^{}=P_{k|k}^{\left(m\right)}. \end{array}$$

### 2.3. Adaptive Iterated Extended Kalman Filter

The EKF overcomes the nonlinear problem by ignoring the higher order terms, which may introduce a truncated error. Thus, the IEKF overcomes this problem. However, it is evident that both the  **Q**  and  **R**  for EKF and those for IEKF are prior estimates. In practice, there are uncertainties in the noise description, and the assumptions on the statistics of disturbances are violated since the availability of precisely known model is unrealistic practical situations. In order to overcome these problems, we employed the noise statistics estimator into the IEKF. Meanwhile, when the system noise is stable and the error variance is small, it is able to employ observation noise estimator only. The corresponding recursive relations are

(10) X^k|k(1)=X^k|k−1,Pk|k(1)=Pk|k−1,Kk(n)=Pk|k−1(H(n))T[H(n)Pk|k−1(H(n))T+R^k−1(n)]−1,X^k|k(n+1)=X^k|k(n)+Kk(n)[yk−h(n)(X^k|k(n))−H(n)  ×(X^k|k−1−X^k|k(n))],Pk|k(n)=[I−Kk(n)H(n)]Pk|k−1(n),

where${\;\;\hat{R}}_{k}^{(n)}$is computed by the time-varying noise statistics estimators with the following equations:

(11) R^k(n)=(1−dk−1)R^k−1(n) +dk−1([I−Hk(n)Kk]υkυkT[I−Hk(n)Kk]T +Hk(n)Pk|k−1(n)(Hk(n))T),

here,  *d*_*k*−1_ = (1 − *b*)/(1 − *b* ^*k*^), 0 < *b* < 1. And then,

(12) $$\begin{array}{c} {\hat{X}}_{k|k}^{}={\hat{X}}_{k|k}^{\left(m\right)},\\ P_{k|k}^{}=P_{k|k}^{\left(m\right)},\\ R_{k}^{}=R_{k}^{\left(m\right)}. \end{array}$$

## 3. Adaptive Iterated Extended Kalman Filter for Integrated Navigation

In this work, we just consider the navigation information for mobile robot in the relative coordinate. The INS error is the accumulation of errors in each time. In order to achieve better estimation accuracy of INS error, the state vector is defined by **x** = [*δP*_*E*_ *δP*_*N*_ *δV*_*E*_ *δV*_*N*_ *δ*Acc_*E*_ *δ*Acc_*N*_]. Here, (*δP*_*E*,*k*_, *δP*_*N*,*k*_), (*δV*_*E*,*k*_, *δV*_*N*,*k*_), and (*δ*Acc_*E*,*k*_, *δ*Acc_*N*,*k*_) are the errors of position, velocity, and accelerometer measured by INS in east and north direction. The system equation for the filter at time *k* is illustrated in.

(13) $$\begin{array}{c} \underset{x_{k}}{\underset{︸}{\left[\begin{array}{c} \delta P_{E,k}\\ \delta {V_{E,}}_{k}\\ \delta \text{Ac}{{\text{c}}_{E,}}_{k}\\ \delta {P_{N,}}_{k}\\ \delta V_{N,k}\\ \delta \text{Ac}{\text{c}}_{N,k} \end{array}\right]}}=\underset{A}{\underset{︸}{\left[\begin{array}{cccccc} 1 & T & T^{2}/2 & 0 & 0 & 0\\ 0 & 1 & T & 0 & 0 & 0\\ 0 & 0 & 1 & 0 & 0 & 0\\ 0 & 0 & 0 & 1 & T & T^{2}/2\\ 0 & 0 & 0 & 0 & 1 & T\\ 0 & 0 & 0 & 0 & 0 & 1 \end{array}\right]}}\underset{x_{k-1}}{\underset{︸}{\left[\begin{array}{c} \delta P_{E,k-1}\\ \delta {V_{E,}}_{k-1}\\ \delta \text{Ac}{{\text{c}}_{E,}}_{k-1}\\ \delta {P_{N,}}_{k-1}\\ \delta V_{N,k-1}\\ \delta \text{Ac}{\text{c}}_{N,k-1} \end{array}\right]}}+W_{k}, \end{array}$$

where *T* is sample time and **W**_*k*_ is the process noise vector. The measurement equation for the filter at time *k* is illustrated in.

(14) $$\begin{array}{c} \underset{y_{k}}{\underset{︸}{\left[\begin{array}{c} \Delta V_{E,k}\\ \Delta V_{N,k}\\ \Delta d_{1,k}^{2}\\ \Delta d_{2,k}^{2}\\ \vdots \\ \Delta d_{m,k}^{2} \end{array}\right]}}=\underset{h\left(X_{k}\right)}{\underset{︸}{\left[\begin{array}{c} \delta V_{E,k}\\ \delta V_{N,k}\\ h_{d_{1}}\left(\delta P_{E,k},\delta P_{N,k}\right)\\ h_{d_{2}}\left(\delta P_{E,k},\delta P_{N,k}\right)\\ \vdots \\ h_{d_{m}}\left(\delta P_{E,k},\delta P_{N,k}\right) \end{array}\right]}}+{\tilde{\upsilon }}_{k}. \end{array}$$

**Figure 1:**
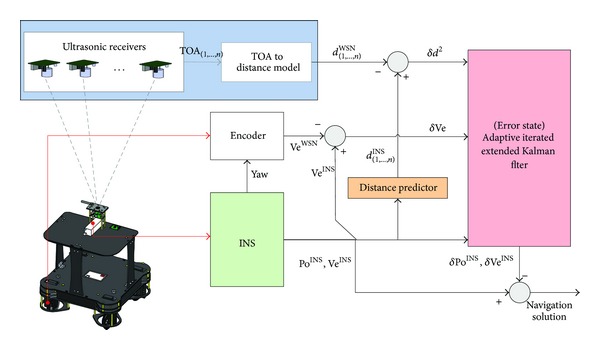
Configuration of the hybrid system.

Here, Δ*d*_*i*_^2^ is the difference between the distance form reference node (RN) to the mobile robot measured by the INS and WSN, respectively, at time *k*, and it is expressed as follows:

(15) Δdi,k2=(diINS)2−(diWSN)2=2(PEINS−PERN,i)δPE,k+2(PNINS−PNRN,i)δPN,k −(δPE,k2+δPN,k2), i=1,2,…,m,

where *d*_*i*_^INS^ and *d*_*i*_^WSN^ are the distances from mobile robot to *i*th RN measured by the INS and WSN, respectively, (*P*_*E*_^INS^, *P*_*N*_^INS^) is INS position for mobile robot, and (*P*_*E*_^RN,*i*^, *P*_*N*_^RN,*i*^) is *i*th RN position. And (Δ*V*_*E*_, Δ*V*_*N*_) is the difference of the WSN and INS velocities in east and north direction, respectively, and ${\tilde{\upsilon }}_{k}$ is measurement noise vector. The derivation of (15) has a very detailed description in [18]. The configuration of the hybrid system is shown in Figure 1.

## 4. Indoor Localization Tests and Discussion

### 4.1. The Architecture of the Integrated Navigation System

**Figure 2:**
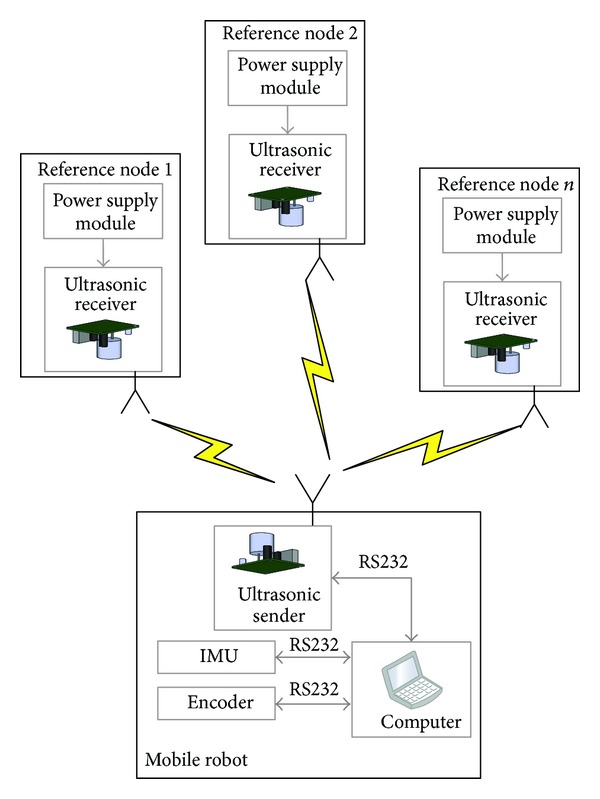
The architecture of the integrated navigation system.

**Figure 3:**
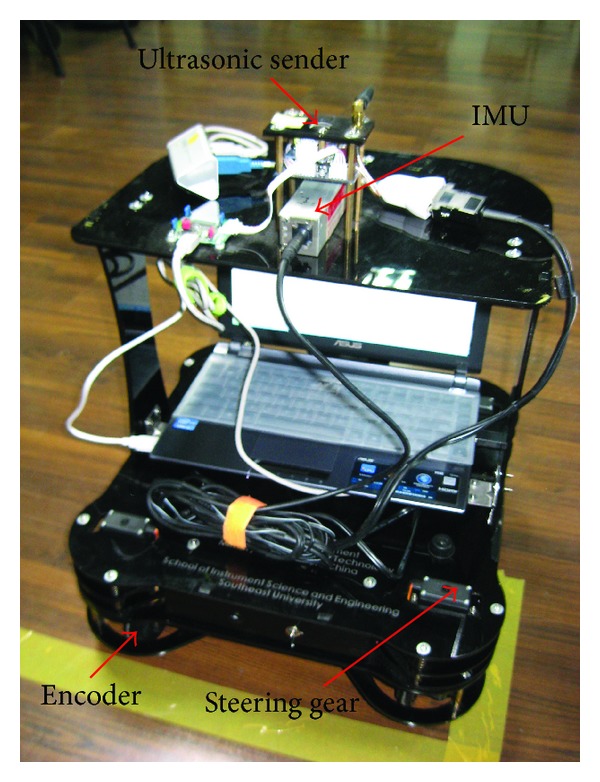
The prototype of the robot.

In this work, a real testbed is built to evaluate the performance of the proposed method.  Figure 2 displays the architecture of the testbed. In this work, the mobile robot (shown in Figure 3) is used to run along the preset trajectory. The IMU fixed to the robot are used to provide INS solution for the position, velocity, and the attitude of the mobile robot. The mobile robot position measured by the WSN is used as ultrasonic sender and the receiver. And the computer is used for saving sensor data.

**Figure 4:**
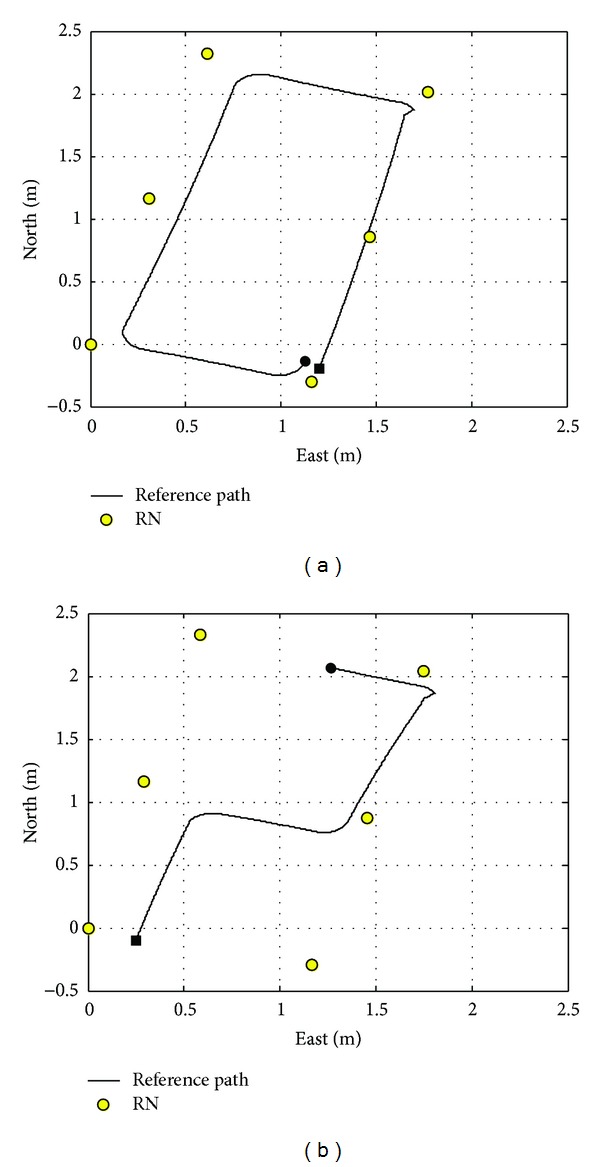
The trajectory of the real test.

The sample time used in the test is 0.02 s, and the mobile robot runs along the trajectories shown in Figure 4 with 0.3 m/s. Meanwhile, the RNs are denoted by yellow circles in Figure 4.

### 4.2. The Performance of the Proposed Method

**Figure 5:**
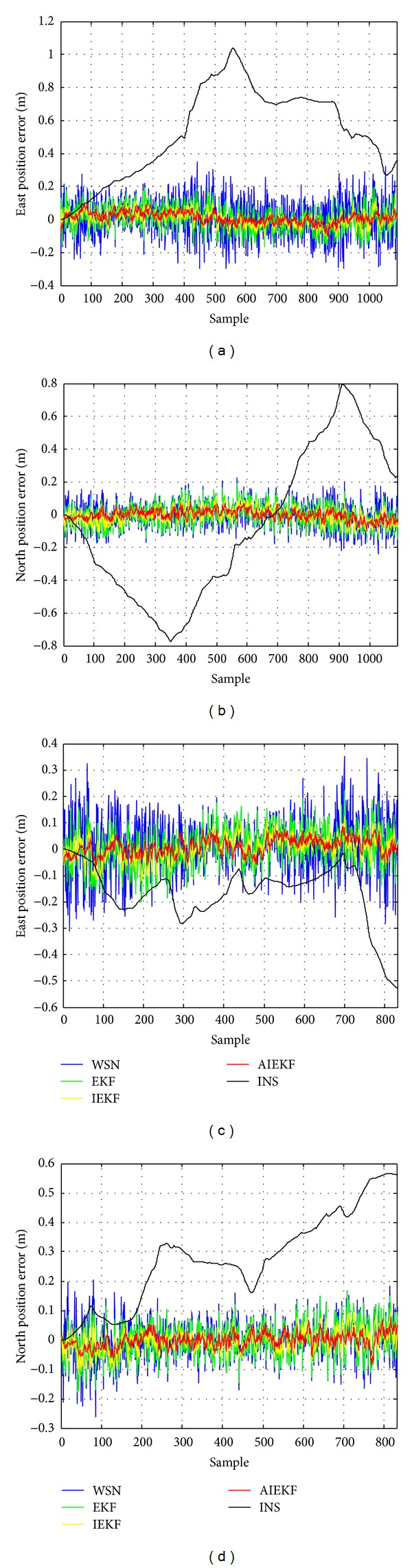
The position errors for the INS only, WSN, EKF, IEKF, and the proposed method. (a) and (b) The first trajectory. (c) and (d) The second trajectory.

In this section, the performance of the proposed method is discussed. The position errors for the INS only, WSN, EKF, IEKF, and the proposed method are shown in Figure 5.

The east and north position errors of five estimation strategies in the first trajectory are shown in Figures 5(a) and 5(b), respectively. From these figures, it can be seen easily that the INS position error is accumulated. WSN is able to maintain the accuracy of position. It is evident that both the EKF and the IEKF are effective in reducing the position error compared with WSN. The errors for the proposed method are smaller than the ones for the IEKF. Figures 5(c) and 5(d) display the east and north position errors of five estimation strategies in the second trajectory. Similar to the first trajectory, it is evident that the proposed method has the smallest error.

The comparison of five estimation strategies in terms of position error is shown in Table 1. The results show that the proposed method has the lowest error in east and north direction respectively. The mean root-mean-square error (RMSE) of position for the proposed method is 0.0295 m. It reduces the mean RMSE of position by about 92.53%, 67.93%, 55.97%, and 30.09% compared with the INS only, WSN, EKF, and IEKF.

Table 2 shows the comparison of five estimation strategies in terms of velocity error. It can be seen that the EKF, IEKF, and the proposed method are able to reduce the velocity error compared with the IN S and the WSN, respectively. The result shows that the mean RMSE of velocity for the proposed method is 0.0468 m/s. However, the velocity estimation accuracy for the EKF, IEKF, and the proposed method is close.

## 5. Conclusions

In this work, the noise statistics estimator is employed into the IEKF to combine the advantages of the AEKF and the IEKF. Then, the AIEKF is used in INS/WSN integrated system. The experimental results show that the proposed method is effective in reducing the position error compared with the INS only, WSN, EKF, and IEKF; however, the velocity estimation accuracy for the EKF, IEKF, and the proposed method is close.

## Figures and Tables

**Table 1:** Comparison of five estimation strategies in terms of position error.

Method	RMSE (m)				
	The first trajectory		The second trajectory		Mean
	East	North	East	North	
INS only	0.5912	0.4590	0.2108	0.3179	0.3947
WSN	0.1132	0.0787	0.1065	0.0697	0.0920
EKF	0.0721	0.0639	0.0736	0.0582	0.0670
IEKF	0.0433	0.0433	0.0462	0.0360	0.0422
The proposed method	0.0333	0.0290	0.0309	0.0249	0.0295

**Table 2:** Comparison of five estimation strategies in terms of velocity error.

Method	RMSE (m/s)				
	The first trajectory		The second trajectory		Mean
	East	North	East	North	
INS only	0.1391	0.1682	0.1400	0.0957	0.1358
WSN	0.0595	0.0854	0.0650	0.0794	0.0723
EKF	0.0441	0.0539	0.0424	0.0437	0.0460
IEKF	0.0425	0.0556	0.0412	0.0482	0.0469
The proposed method	0.0445	0.0546	0.0420	0.0462	0.0468
